# Integral Electron Scattering Cross Sections from N_2_O for Impact Energies Ranging from 1 to 1000 eV

**DOI:** 10.1021/acs.jpca.3c07708

**Published:** 2024-01-16

**Authors:** Ana I. Lozano, Jaime Rosado, Francisco Blanco, Paulo Limão-Vieira, Gustavo García

**Affiliations:** † Instituto de Física Fundamental, Consejo Superior de Investigaciones Científicas, Serrano 113-bis, 28006 Madrid, Spain; ‡ Laboratório de Colisões Atómicas e Moleculares, Departamento de Física, CEFITEC, Universidade NOVA de Lisboa, 2829-516 Caparica, Portugal; § Institut de Recherche en Astrophysique et Planétologie (IRAP), Université Toulouse III − Paul Sabatier, 9 Avenue du Colonel Roche, 31028 Toulouse, France; ∥ Departamento de Estructura de la Materia, Física Térmica y Electrónica e IPARCOS, 16734 Universidad Complutense de Madrid, Avenida Complutense, E-28040 Madrid, Spain

## Abstract

Accurate total cross sections (TCS), within 5%, for electron scattering by N_2_O molecules have been measured with a magnetically confined electron transmission apparatus for impact energies ranging from 1 to 200 eV. For higher energies, these measurements have been complemented with our independent atom-based screening corrected additivity rule, including interference (IAM-SCAR + I) method to determine a complete reference TCS data set in the energy range (1–1000 eV). After a critical discussion that includes our calculated integral elastic and ionization cross sections and the theoretical and experimental data available in the literature, a complete set of integral elastic and inelastic (rotational, vibrational, and electronic excitation, ionization and electron attachment) cross sections, consistent with the reference TCS data, have been derived. This update on the N_2_O collisional database may help to improve the accuracy of radiation-induced transport models.

## Graphical abstract

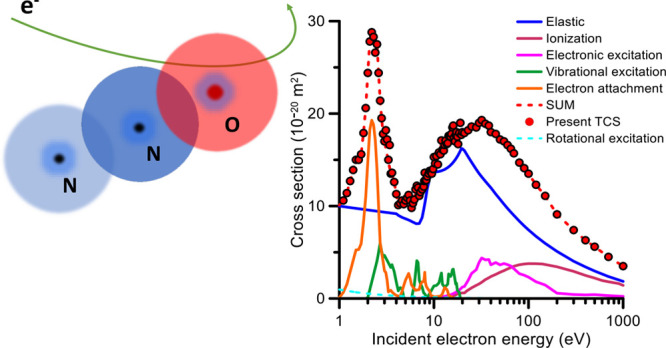

## 1 Introduction

Nitrogen oxides are reactive molecular species with relevant effects in the biosphere. Nitric oxide (NO) and nitrogen dioxide (NO_2_) are generally produced by fossil fuel combustion in the presence of nitrogen and severely contribute to air pollution, yielding toxic effects on living organisms. Nitrous oxide (N_2_O) is relatively less reactive; it has been used in the industry and for medical treatments as an anesthetic but also contributes to biosphere alterations due to its impact on ozone depletion and global warming, with an estimated residence time of 150 years.^1^ The landmark United in Science report notes N_2_O global atmospheric concentration in 2017 of 329.9 ± 0.1 ppb, meaning 122% of preindustrial levels.^1^ The relevance of these nitrogen oxides in atmospheric models motivated a significant number of experimental and theoretical studies devoted to electron scattering cross section determinations.

Early total electron scattering cross sections (TCS) from N_2_O were performed with the help of Ramsauer-type apparatuses.^2,3^ Further TCS measurements were published by Szmytkowski et al.,^4–6^ Kwan et al.,^7^ Zecca et al.,^8^ Shilin et al.,^9^ and Pyun et al.^10^ for different impact energies, covering a full range from 0.4 up to 4250 eV. In order to derive a complete set of electron scattering cross section data, these results, together with other experimental and theoretical elastic and inelastic integral cross sections available in the literature, have been compiled and discussed by different authors.^11–13^ The most recent review of Song et al.^13^ presents a comprehensive set of recommended data, which constitutes the main motivation for the present study.

As pointed out in previous articles,^14,15^ accurate TCS values are essential to determine a self-consistent electron scattering data set for modeling purposes. TCS are reference data to check the consistency of the integral cross sections assigned to each open scattering channel at a given incident electron energy. In addition, if the energy resolution allows experimental evidence of resonant features, then experimental TCSs can be used to confirm predicted resonances by different theories and even those from electron transmission experiments. For this reason, the first objective of this study is to obtain a reliable set of electron scattering TCS from N_2_O over a broad energy range (1–1000 eV). From 1 to 200 eV, TCS has been measured with a “state of the art” magnetically confined electron transmission apparatus^16^ with a total uncertainty limit within 5%. For higher energies, TCS values have been complemented with our independent atom screening corrected additivity rule, including interference effects (IAM-SCAR + I) method,^17^ thus obtaining a complete set of reference data from 1 to 1000 eV. As described in the next sections, through a critical analysis of available data together with our calculated integral elastic and ionization cross sections, recommended integral cross sections for all the scattering channels allowed in this energy range are presented in this study.

The remainder of this article is organized as follows: the experimental and theoretical methods used in this study are described in Section 2, the results are presented and discussed in Section 3, and the conclusions that can be drawn are in Section 4.

## 2 Experimental and Theoretical Methods

Electron scattering TCSs have been measured with the experimental system described elsewhere.^16^ Basically, it consists of a pulsed linear transmission beam apparatus with strong axial magnetic field (0.1 T) confinement. Under these conditions, we can assume that every scattering event is converted into a kinetic energy loss in the forward direction, which can be measured with a retarding field analyzer (see ref 16 for details). Note that any electrons scattered at angles higher than 90° move back to the cathode, where they are again reflected toward the interaction region. The electron beam is produced by an emitting thermionic tungsten filament and focused along the magnetic field axis onto the entrance aperture of a gas cell containing a sample of molecular nitrous oxide. This chamber is a cooling electron trap, where the energy spread of the beam (Δ*E*) is reduced to 100 meV through successive collisions with N_2_ molecules. The primary electron beam is then pulsed and accelerated/decelerated to the required incident energy (*E*) at the entrance aperture (1.5 mm in diameter) of the scattering chamber (SC), which contains the target molecules (N_2_O in this case) at a well-known gas pressure. A 1.5 mm exit aperture defines the collision length (*L* = 40 mm). The target gas is introduced into this chamber through a leak valve, where it is maintained at a constant pressure, as measured with an MKS-Baratron 627B absolute capacitance manometer. The gas pressure was varied from 0 to 6 mTorr during the measurements, and the transmitted intensity was recorded for at least five different pressure values within this range. Electrons emerging from the SC are energy-selected by a retarding potential energy analyzer (RPA), and finally detected by a double microchannel plate electron multiplier operating in a single counting mode.

The experimental electron total scattering cross section (σ_t_) is obtained for each incident electron energy from the Beer–Lambert attenuation law:

$$I=I_{0}e^{‐n\sigma_{t}L}$$ 1

where *I* is the transmitted electron intensity, *I*_0_ is the initial intensity when there is no gas in the SC, *n* is the N_2_O gas density, and *L* is the length of the collision chamber. Assuming an ideal gas behavior, eq 1 can be written as

$$\ln(\frac{I}{I_{0}})=‐L\sigma_{t}n=\frac{Lp}{kT}\sigma_{t}$$ 2

In eq 2, *k* is Boltzmann’s constant, *T* is the absolute temperature, and *p* is the N_2_O gas pressure. *T* is derived from *T* = $\sqrt{T_{c}T_{m}}$ , where *T*_c_ is the temperature of the collision chamber (measured with a calibrated thermocouple) and *T*_m_ is the temperature of the Baratron manometer. From the operation conditions of this experiment, *T*_c_ ≈ *T*_m_ and therefore thermal transpiration effects are practically negligible. The accuracy on the pressure measurements is assumed to be better than 1%, as stated by the manufacturer. The entire measurement conditions, data acquisition, and data analysis are monitored and controlled by a custom-designed LabView (National Instruments) program.

From the different sets of experiments, the attenuation of the electron beam passing through the SC containing different pressures of N_2_O for different impact energies (2, 20, and 100 eV) is shown in Figure 1.

**Figure 1:**
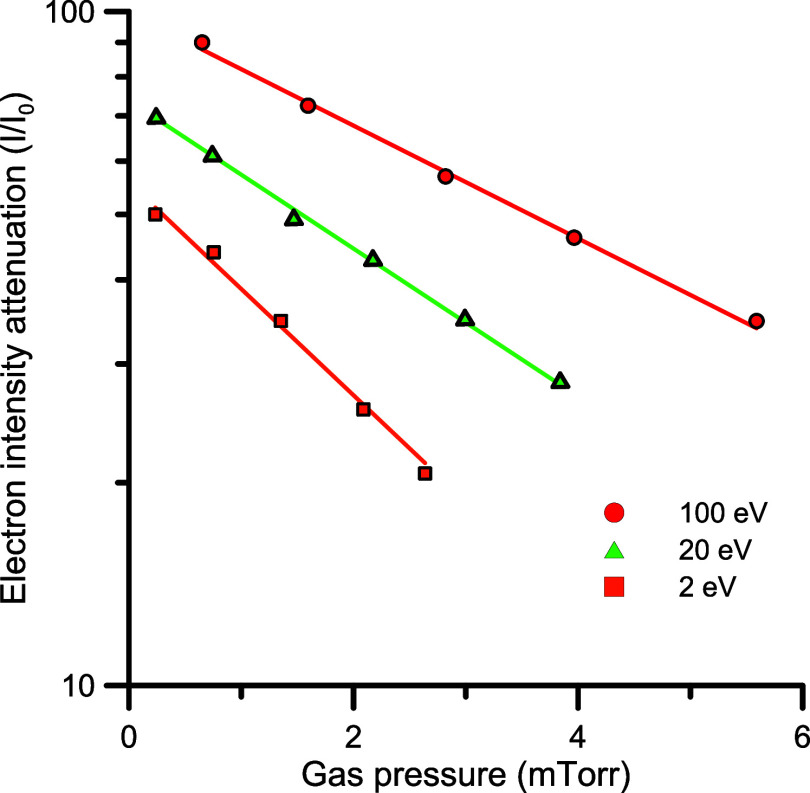
Attenuation of the electron beam passing through the scattering chamber containing different pressures of N_2_O for different impact energies (see legend in figure).

The total experimental uncertainty limits on these measurements are within 5%, as derived by adding in quadrature all the known uncertainty sources, i.e., a statistical uncertainty (our measurement procedures were repeated at least 5 times to ensure standard deviations below 4.5%), the uncertainty in the pressure measurement (1%), energy calibrations (1%), and the scattering length determination (1.5%). From the conditions of this experiment, the magnetic beam intensity along the SC ensures a cyclotron radius of the colliding electron that is less than 0.5 mm for the whole energy range considered here (1–200 eV). This means that the effective diameter of the electron beam in the SC is lower than the entrance and exit aperture diameters, ensuring that no collimating effects are distorting the present measurements. For the low-pressure conditions of those measurements, eq 2 was fitted to a single exponential function (see Figure 1), with 0.999 correlation index thus indicating that multiple scattering effects are not contributing to the present results. Transmitted intensities, for N_2_O pressures ranging from 0 to 6 mTorr, typically varied from 2 × 10^3^ to 0.7 × 10^2^ electrons/second, which corresponds to equivalent electron currents from 3 × 10^–16^ to 1.1 × 10^–17^ A. For such low current conditions, no dependence of the cross sections on the electron current was observed, which indicates that space charge effects are not present.

In addition, transmission beam TCS measurements present a systematic error due to the acceptance angle of the detector (missing angle). Electrons elastically or rotational inelastically scattered into these angles are accounted as unscattered and therefore tend to lower the magnitude of the measured TCS. For magnetically confined experimental systems this systematic error depends on both the incident energy and Δ*E*. This effect is discussed in detail in ref 16 and can be corrected by integrating the calculated differential cross sections over the corresponding missing angles.

As aforementioned, total scattering and ionization cross sections have been calculated with our independent atom with screening corrections and interference effects (IAM-SCAR + I) method.^17–20^ This is a well-established procedure that has been demonstrated to be reliable within 10%, at impact energies above 20 eV, for a large number of molecular targets, from triatomic^21^ to more complex molecules.^22^ Hence, we present here the results of this calculation only for impact energies above 10 eV.

N_2_O is a linear molecule, and due to the geometrical positions of its constituent atoms (see Figure 2), has a weak permanent dipole moment (0.316 D) with an averaged rotational excitation energy of 2.14 meV at room temperature. In order to include the rotational excitation cross sections in the theoretical description of the molecule, we performed an independent calculation by assuming the molecule as a rigid rotor and applying the first-order Born approximation in combination with Dickinson’s correction.^23^

**Figure 2:**
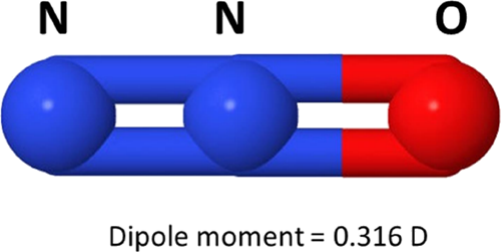
Geometrical configuration of the N_2_O molecule.

## 3 Results and Discussion

### 3.1 Total Electron Scattering Cross Section

The present experimental results of the total electron scattering cross sections from N_2_O molecules are shown in Table 1 and plotted in Figure 3.

**Table 1:** Total Electron Scattering and Integral Cross Section Measured and Calculated in This Study (All in SI Units)

**energy (eV)**	**experiment**		**calculation**			
	TCS (10^–20^ m^2^)	uncertainty (±)	TCS (10^–20^ m^2^)	elastic (10^–20^ m^2^)	ionization (10^–20^ m^2^)	rotational excitation (10^–20^ m^2^)
1.1	10.6	0.3				0.95
1.2	11.4	0.2				
1.3	12.7	0.3				
1.4	13.9	0.6				
1.5	15.0	0.2				0.67
1.6	15.0	0.5				
1.7	15.6	0.4				
1.8	17.9	0.6				
1.9	20.6	0.6				
2.0	24.4	0.5				0.53
2.1	27.8	0.7				
2.2	28.8	0.7				
2.3	28.3	0.6				
2.4	26.6	0.9				
2.5	27.5	1.0				
2.6	24.4	0.7				
2.7	21.4	0.7				
2.8	19.5	0.4				
2.9	17.8	0.4				
3.0	17.0	0.3				0.37
3.1	18.2	0.6				
3.2	17.2	0.4				
3.3	15.0	0.3				
3.4	16.6	0.5				
3.5	13.5	0.5				
3.6	13.6	0.5				
3.8	12.6	0.5				
4.0	11.3	0.3				0.29
4.2	10.1	0.4				
4.4	10.3	0.3				
4.6	10.6	0.3				
4.8	10.2	0.4				
5.0	10.6	0.1				0.23
5.2	10.9	0.4				
5.4	11.2	0.2				
5.6	10.6	0.4				
5.8	9.8	0.4				
6.0	10.3	0.3				
6.2	10.7	0.2				
6.4	11.8	0.4				
6.6	12.2	0.4				
6.8	12.0	0.2				
7.0	11.1	0.3				0.17
7.2	11.5	0.2				
7.5	11.8	0.5				
7.8	12.1	0.4				
7.9	12.7	0.3				
8.0	13.6	0.3				
8.1	12.8	0.5				
8.3	12.8	0.2				
8.5	13.2	0.5				
8.8	13.5	0.5				
9.0	13.8	0.5				
9.3	13.7	0.5				
9.5	13.5	0.1				
9.8	14.0	0.4				
10.0	15.3	0.4	19.9	19.9		0.13
10.3	14.6	0.2				
10.6	15.4	0.5				
11.0	14.5	0.2				
11.3	15.8	0.2				
11.7	15.6	0.2				
12.0	17.1	0.4				
12.3	16.1	0.6				
12.7	16.0	0.6				
13.0	16.6	0.5				
13.3	17.1	0.6				
13.7	16.7	0.4				
14.0	16.8	0.4				
14.3	17.6	0.5				
14.7	17.5	0.7				
15.0	17.5	0.4	18.7	18.3	<0.01	0.087
15.3	17.8	0.5				
15.7	18.5	0.7				
16.0	18.7	0.1				
16.3	17.4	0.5				
16.7	17.0	0.2				
17.0	17.7	0.4				
17.3	18.0	0.7				
17.7	17.5	0.5				
18.0	16.7	0.3				
18.5	17.9	0.7				
19	19.0	0.4				
19.5	17.7	0.6				
20	18.0	0.3	18.1	16.4	0.48	0.067
21	17.9	0.6				
23	18.2	0.5				
25	18.6	0.6				
28	18.5	0.4				
30	19.0	0.50	17.6	13.5	2.2	0.047
32	19.3	0.4				
35	18.9	0.3				
38	18.7	0.3				
40	18.0	0.5	16.8	12.0	3.2	0.036
45	17.8	0.4				
50	17.2	0.3	15.8	10.7	3.6	0.029
55	17.0	0.4				
60	16.6	0.5				
65	16.2	0.4				
70	15.7	0.4	14.1	9.0	3.9	0.021
80	14.7	0.4				
90	14.1	0.3				
100	13.5	0.2	12.3	7.4	3.8	0.016
120	12.3	0.3				
150	11.1	0.3	10.4	6.0	3.5	0.011
200	9.0	0.5	9.1	5.1	3.2	<0.01
300			7.4	4.0	2.7	
400			6.3	3.4	2.3	
500			5.6	3.0	2.0	
700			4.5	2.4	1.6	
1000			3.5	1.8	1.3	

**Figure 3:**
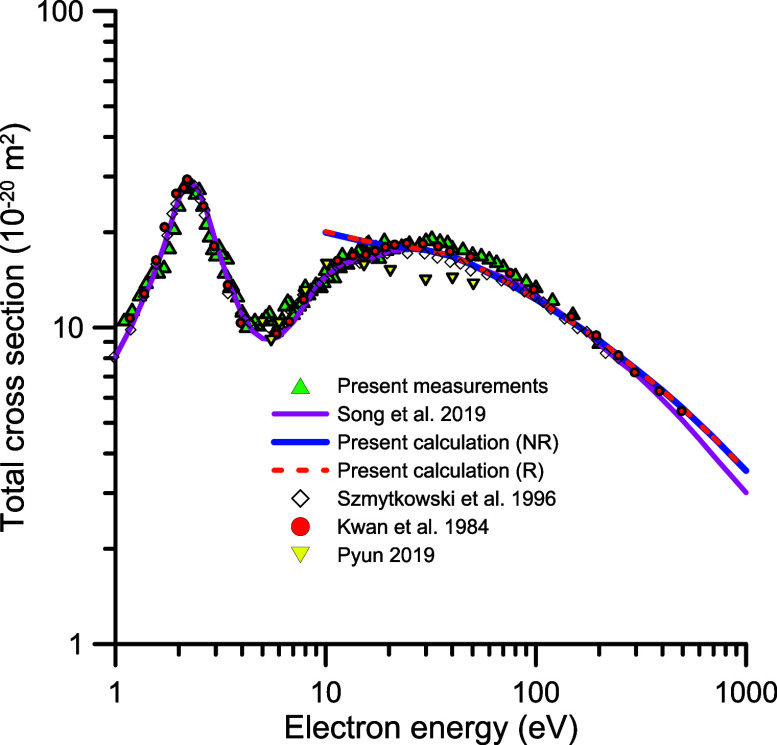
Total electron scattering cross section by N_2_O: green triangle, present measurements; red circle, Kwan et al.^7^; ◇, Szmytkowski et al.^5^; inverted orange triangle, Pyun et al.^10^; blue line, present calculation (excluding rotational excitations-NR); red dashed line, present calculation (including rotational excitation-R); purple line, recommended values of Song et al.^13^

Comparing the present TCS measurements and calculations, for energies above 10 eV there is a general agreement within the estimated uncertainty limits (see Figure 3). The experimental values tend to be higher than our IAM-SCA + I calculation in the range of 20–50 eV, which is an indication of the prevalence of the molecular structure of N_2_O even at such relatively high impact energies. In order to compare our experimental data with previous measurements and the recommended values of Song et al.,^13^ Figure 4 shows these results from 1 to 100 eV in a linear scale. A close inspection of the figure shows an excellent agreement as far as the shape and position of the ^2^Π resonance are concerned between 1 and 4 eV with experimental data of Szmytkowski et al.,^4–6^ Kwan et al.^7^ and the recommended values of Song et al.^13^ From 4 to 10 eV the present experimental data also reveal some resonant features, which will be discussed later. For energies above 10 eV, data from refs 6,10, and 13 tend to be lower in magnitude than the present ones, reaching a maximum discrepancy of about 11–35% at 30 eV. These discrepancies are probably connected with the energy spread of the primary beam and the energy and angular resolution of the detector used by the different experimental arrangements. However, the older results from Kwan et al.^7^ show an excellent agreement with ours, within the uncertainty limits. For higher energies, above 100 eV, all of the experimental results tend to converge (Figure 3) among themselves.

**Figure 4:**
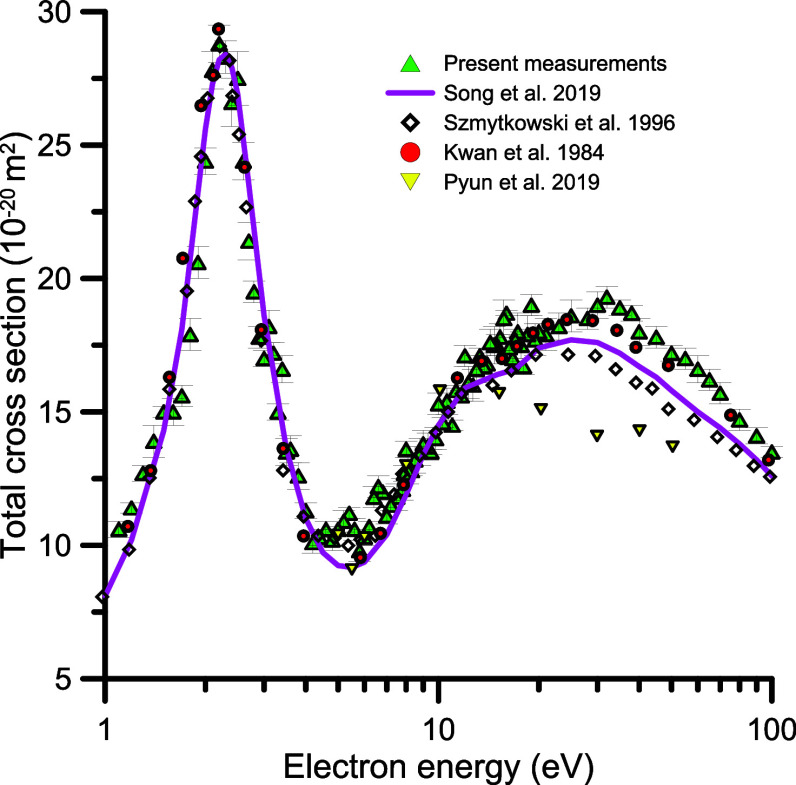
Total electron scattering cross section for low and intermediate electron impact energies (1–100 eV). See also the legend in Figure 3.

The result of combining our experimental TCS data from 1 to 200 eV with our calculation in the energy range of 200–1000 eV is plotted in Figure 5 and, as already mentioned, will be considered as reference data to check the consistency of the integral cross section data assigned to the different scattering channels (see Subsections [Sec sec3.2], 3.3, 3.4).

**Figure 5:**
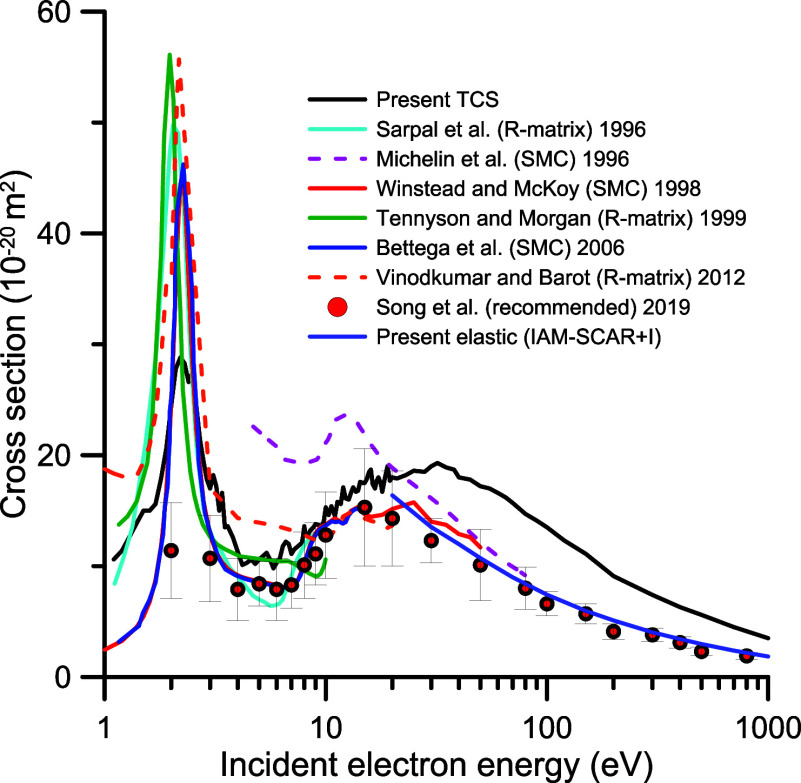
Integral electron scattering cross sections from N_2_O. **—**, present combined experimental and theoretical total electron scattering cross sections; **—**, present calculated elastic scattering cross section (IAM-SCAR + I method); red line, calculated elastic cross sections (SMC method) of ref 24; blue line, elastic scattering SMC calculations from ref 25; purple dashed line, elastic scattering SMC calculations from ref 26; light blue line, elastic scattering R-matrix calculations from ref 27; green line, elastic scattering R-matrix calculations from ref 28; red dashed line, elastic scattering R-matrix calculations from ref 29; red circle, elastic scattering cross sections recommended in ref 13.

### 3.2 Integral Elastic Cross Section

Different calculated integral elastic cross section sets available in the literature are plotted in Figure 5, together with our present calculation (above 20 eV). As we mentioned in a recent review on the CO_2_ molecule,^21^ for the integral elastic, we recommend on the information from the calculations. The combination of accurate “ab initio” low-energy scattering methods with intermediate-high energy model potential calculations provides, in general, integral elastic cross sections with verified uncertainties within 10% (see, e.g., refs 21 and 22 However, experimental integral elastic cross section (IECS) values are derived from the integration of the corresponding differential elastic cross section measurements, which are in general contaminated with the rotational excitation and require theoretical extrapolations to both small and large scattering angles, leading, in general, to higher uncertainty limits (20–25%). In addition, the absolute experimental DCS values require normalization procedures based on relative flow techniques^2^ or directly from calculations. A detailed comparative analysis of the available experimental IECS results can be found in Song et al.^13^

For the lower energies considered in this study (1–20 eV), previous calculations qualitatively agree but they show discrepancies in the exact position of the ^2^Π resonance and the absolute values of the IECS (see Figure 5).

Taking our TCS values as reference data, we find that the calculation of Winstead and McKoy,^24^ using the Swinger multichannel (SMC) method, shows an excellent agreement on the position of this resonance with that experimentally observed. McKoy’s results were extended by Bettega et al.^25^ up to 50 eV, maintaining the concordance with the present TCS. However, R-matrix and SMC calculations from Vinodkumar and Barot^29^ and Michelin et al.^26^ give IECS values higher in magnitude than the present TCS, in contradiction with our reference data. Other R-matrix calculations from Morgan et al.,^30^ Tennyson and Morgan^28^ and Sarpal et al.^27^ agree with our reference TCS data but tend to place the energy position of the ^2^Π resonance below the experimental result. For electron energies from 20 to 50 eV, our calculated IECSs provide reliable data, within 10%, and show a good agreement with the calculations of Winstead and McKoy^24^ and Bettega et al.,^25^ within the mentioned uncertainty limits. The recommended IECS values of Song et al.^13^ are mainly based on experimental data and, considering their quoted uncertainty, agree well, in general with the most representative calculations (see ref 13 for details). Note that in the energy range of 2–4 eV, the recommended IECS do not include the prominent ^2^Π resonance. This is an appropriate interpretation since this shape resonance is due to the temporary trapping of the incident electron by the effective potential. Although these resonances appear in the elastic scattering calculation, in fact, these processes are inelastic and should be treated as electron attachment processes. Note that the electron detachment process after the temporary anion formation has a finite probability, but we here refer to elastic processes those in which only kinetic energy is transferred to the target. From the above considerations we propose as recommended integral elastic cross sections the low-intermediate energy (1–20 eV) SMC calculations from refs 24 and 25 (excluding the resonances which will be accounted as electron attachment processes) in combination with our higher energy (20–1000 eV) IAM-SCAR + I calculation with an overall uncertainty of about 10%.

### 3.3 Ionization Cross Sections

The total ionization cross sections (TICSs) available in the literature^31–33^ agree very well each other, within 5% (see Figure 6), except for the old measurements from Rapp and Englander-Golden.^34^ We note that in refs 31–33 the TICSs are the result of adding the partial cross sections assigned to the single charged cationic species detected with a time-of-flight (TOF) mass spectrometer, thus they really represent the total single ionization cross section. In contrast, results from ref 34 are related to the total induced positive ion current, i.e., they represent the TICS but somehow are overestimated due to the contribution of multiple charged ions. Apart from the expected disagreement at the lower energies, our TICS calculation agrees reasonably with the three more recent sets of experimental data.^31–33^ We can then conclude that the total single ionization cross section included in our self-consistent data set is based on that experimentally determined in refs 31–33 with an estimated uncertainty limit of about 7%. Note that double ionization is not considered in this study. Since, in the energy range considered here, multiple ionization cross sections are about 1 order of magnitude lower than single, such contribution lies within the assigned uncertainty limit. However, special precautions must be taken for specific applications focused on energy loss and stopping powers, where multiple ionization processes become relevant for increasing energies.^35^

**Figure 6:**
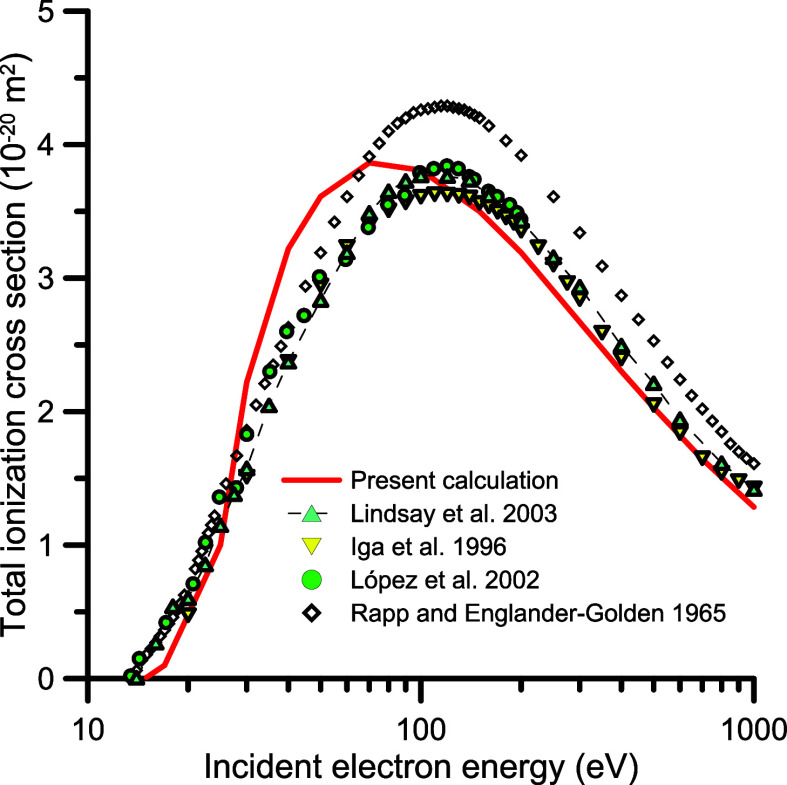
Total ionization cross sections. ◇, ref 34; blue triangle, ref 33; orange inverted triangle, ref 31; green circle, ref 32; red line, present calculation.

Although the integral cross section analysis does not intend to reach the level of describing the induced (positive, negative, and neutral) fragmentation by electron impact, it is interesting to note that most of the references cited in Figure 6 provide partial ionization cross section (PICS) data for positive fragments. A complete summary of these results is found in Song et al.’s^13^ review. Nonetheless, and in order to contribute to the analysis of the cationic fragmentation for electron impact in the energy range of 13–1000 eV, we have used our data to derive the PICSs by combining the present TICS calculation with the relative cation intensity as measured with a standard TOF mass spectrometer (see ref 36 for details on the experimental setup). A typical mass spectrum for the 70 eV electron impact energy is shown in Figure 7. The combination of branching ratios with the calculated TICS provides the PICS values shown in Figure 8 from the ionization threshold up to 1000 eV

**Figure 7:**
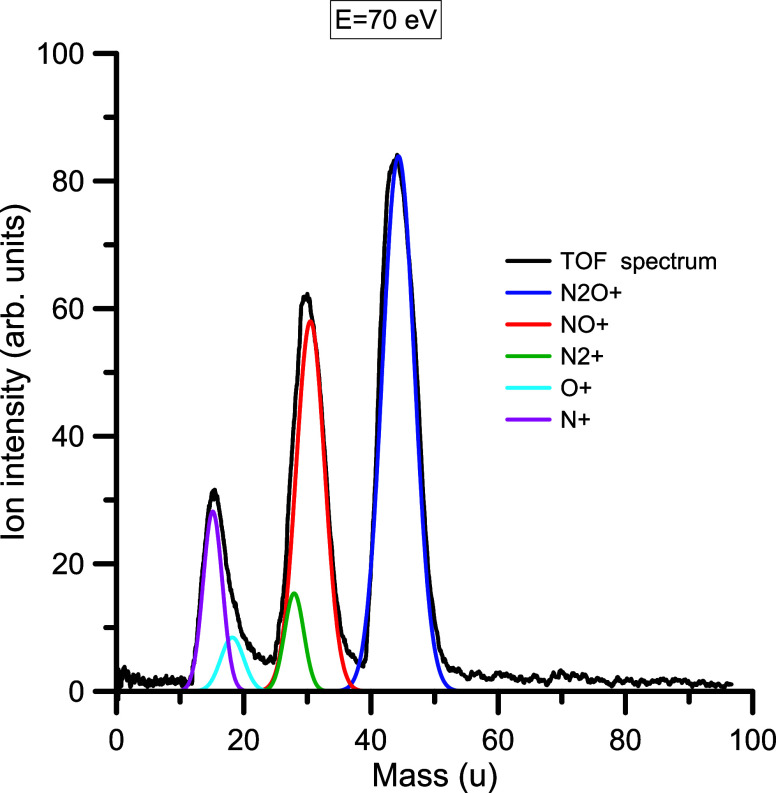
TOF mass spectrum of the cationic fragmentation of N_2_O produced by a 70 eV electron impact. See ref 36 for details on the experimental setup.

**Figure 8:**
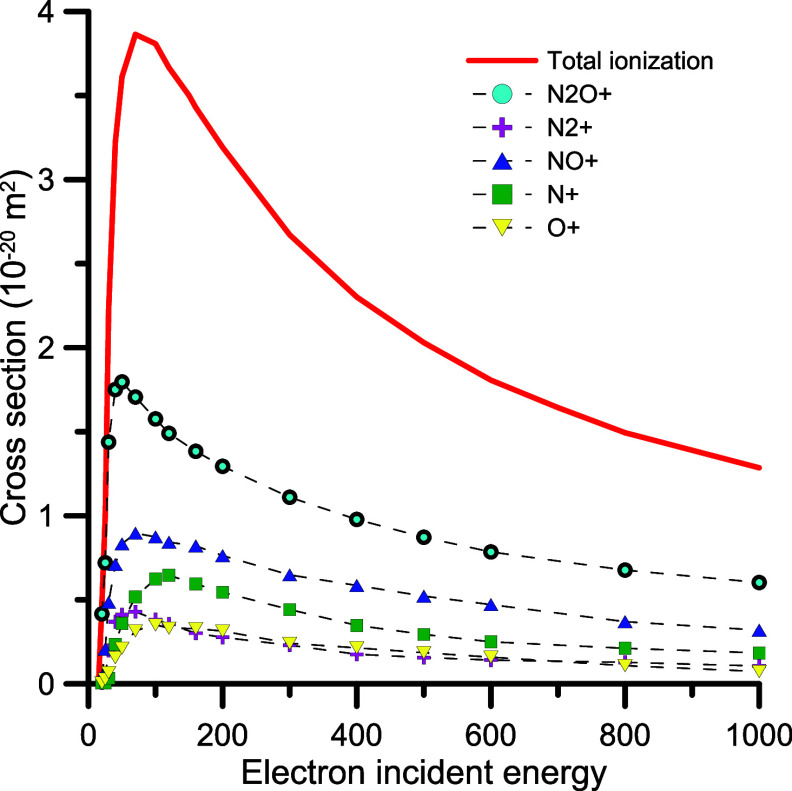
Present total and partial ionization cross sections derived with the semiempirical procedure described in Section 3.3.

### 3.4 Other Inelastic Channels

As already mentioned, from a theoretical point of view, electron attachment cross sections appear as resonances superimposed to the calculated integral elastic cross sections. According to the procedure, we proposed to derive a complete set of inelastic cross sections,^21^ the electron attachment cross section is derived by subtracting the integral elastic cross section (once removed the calculated resonances) from the experimental TCS reference values. All the resonant structures around the anion formation peaks reported by Rapp and Briglia^37^ and Krishnakumar and Srivastava^38^ have been assigned to electron attachment processes. As shown in Figure 9, the agreement in the position of the resonances is remarkable, and the magnitude of the electron attachment cross sections is consistent with the observed anion current. It is larger than the corresponding anion formation current but this can be expected since the subsequent electron detachment channel is always open.

**Figure 9:**
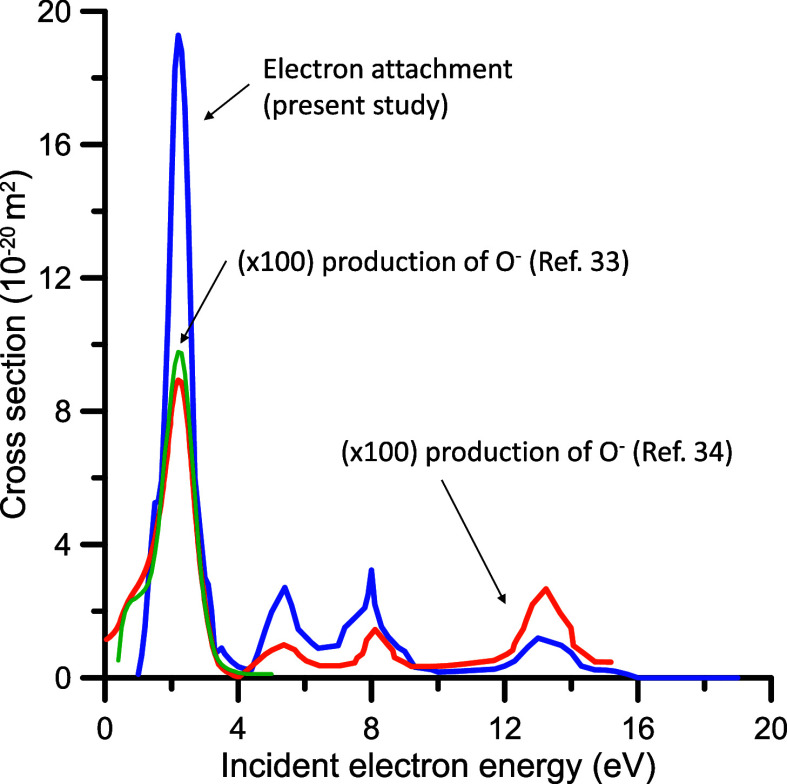
N_2_O electron attachment cross sections. **—**, present study. O^–^ production measured by **—**, Rapp and Briglia;^37^ **—**, Krishnakumar and Srivastava.^38^

Above the electronic excitation threshold, most of the nonionizing collisions have been accounted as electronic excitations, and the remaining low-energy cross sections have been considered as vibrational excitations. Since the latter have been derived from the subtraction of comparatively high numerical values, their associated uncertainties are much higher than those of the former channels. Nonetheless, according to the present self-consistent procedure, we estimate these uncertainties to be about 20%.

The partition of the TCS into the elastic and the different inelastic channels is shown in Figure 10 with the corresponding values listed in Table 2. Note that the assigned cross section values to each single scattering channel (elastic, ionization, electronic excitation, vibrational excitation, and electron attachment) agree well with the corresponding available information.^24,25,32,33,37–39^ In particular, the remaining vibrational excitation cross sections present a peak structure in concordance with that reported by Allan and Skalický^39^ for the excitation of the (000), (001), (100), (010), and (200) vibrational modes of the N_2_O molecule. For completeness, we have also included in Figure 10 our calculated rotational excitation cross section. Note that, due to its weak dipole moment, rotational excitation cross sections are negligible with respect to those of the other scattering channels considered in this study. Finally, the consistency of the proposed data set is supported by the shape of the summed values with the present TCS reference data.

**Figure 10:**
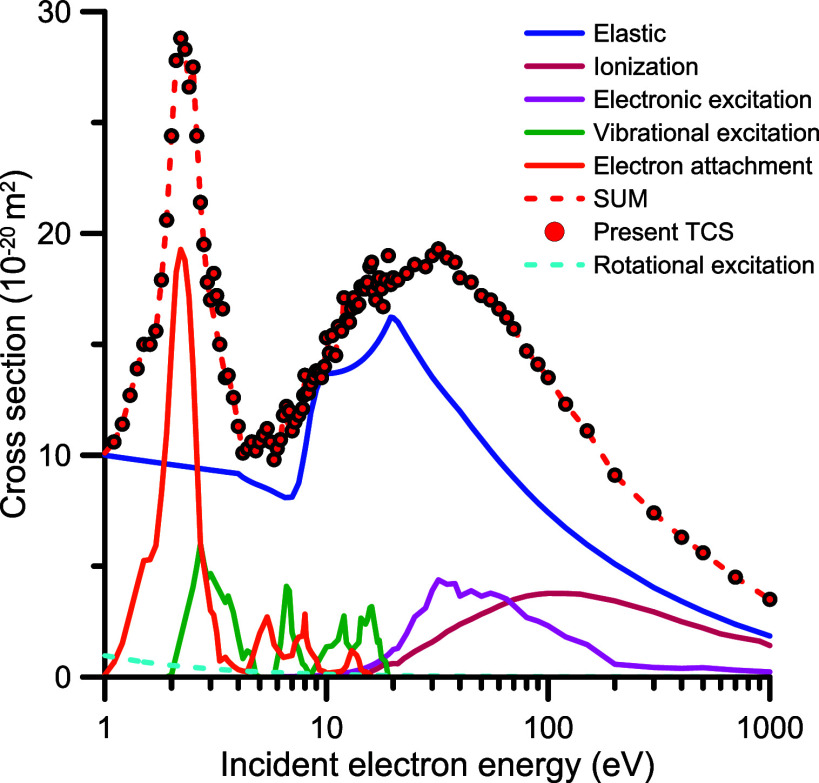
Recommended integral electron scattering cross sections from N_2_O: blue line, elastic; red line, ionization; purple line, electronic excitation; green line, vibrational excitation; orange line, electron attachment; blue dashed line, rotational excitation. The sum of the cross sections of all these scattering channels (red dashed line) is also compared to the present recommended total cross sections (red circle).

**Table 2:** Recommended Integral Cross Sections for All of the Elastic and Inelastic Channels Available in the Electron Impact Energy Range (1–1000 eV) (All in SI Units)

*E* (eV)	elastic	rotational excitation	vibrational excitation	electronic excitation	electron attachment	ionization
1	10.0	0.848			0.100	
1.5	9.75	0.5992			5.25	
2	9.58	0.467	0.1		14.8	
3	9.34	0.328	4.66		3.00	
4	9.17	0.256	1.79		0.335	
5	8.63	0.210			1.97	
7	8.11	0.156	2.0	0.0217	0.968	
10	13.7	0.114	1.36	0.0860	0.175	
15	14.7	0.0792	2.39	0.409	0.235	0.045
20	16.4	0.0613		1.239		0.603
30	13.5	0.0426		3.90		1.57
40	12.0	0.0328		3.64		2.37
50	10.7	0.0268		3.64		2.84
70	8.99	0.0197		3.22		3.49
100	7.42	0.0142		2.31		3.78
150	5.96	0.00983		1.46		3.67
200	5.10	0.00756		0.576		3.43
300	4.03	0.00521		0.432		2.94
400	3.46	0.00400		0.430		2.49
500	2.97	0.00325		0.420		2.21
700	2.38	0.00238		0.312		1.81
1000	1.85	0.00171		0.229		1.42

## 4 Conclusions

Accurate total electron scattering cross sections, within 5%, have been measured with a “state of the art” magnetically confined electron transmission apparatus for impact energies in the range of 1–200 eV. For the lower energies (1–10 eV), excellent agreement is found with previous measurements and the recommended data of Song et al.^13^ in terms of magnitude of the TCS and position of the shape (^2^Π) resonance at 2.2 eV. For intermediate energies (4–100 eV), although a general agreement within the combined uncertainty limits is found, the present results tend to be higher in magnitude (up to 7%) showing some resonant features (not reported previously), which locally increase this disagreement up to 15%. These discrepancies were assigned to the angular and energy resolution of the different experimental arrangements. For energies between 10 and 100 eV, we have calculated the electron scattering TCS by using our IAM-SCAR + I method, which is considered reliable within 10% for impact energies above 20 eV. Calculated values agree with the present measurement in the overlapping energy range (10–200 eV), yet above 300 eV tend to be higher than that recommended by Song et al.^13^ reaching a maximum discrepancy of 16% at 1000 eV. We have considered our TCS measurements (1–200 eV) complemented with our calculated values (200–1000 eV) as reference data to derive a complete set of self-consistent integral scattering cross sections for e-N_2_O collisions in the electron impact energy range (1–1000 eV). According to the critical discussion described in the previous section, including our calculated integral elastic and ionization cross section and the theoretical and experimental data available in the literature, we have derived a complete set of integral elastic and inelastic (rotational, vibrational, electronic excitation, ionization, and electron attachment) cross sections, which are consistent with our recommended TCSs. Since N_2_O is a relevant atmospheric molecule for which electron scattering cross section data are being demanded by international organizations, such as EURAMET,^40^ in order to establish accurate radiation-induced particle transport models in the biosphere and their consequences in human health, we consider this update of the N_2_O collisional database may help improving the accuracy of these models.
